# Physiologic responses to a staircase lung volume optimization maneuver in pediatric high-frequency oscillatory ventilation

**DOI:** 10.1186/s13613-020-00771-8

**Published:** 2020-11-18

**Authors:** Pauline de Jager, Johannes G. M. Burgerhof, Alette A. Koopman, Dick G. Markhorst, Martin C. J. Kneyber

**Affiliations:** 1 Department of Paediatrics, Division of Paediatric Critical Care Medicine, Beatrix Children’s Hospital, University Medical Center Groningen, P.O. Box 30.001, 9700 RB Groningen, The Netherlands; 2 Department of Epidemiology, University Medical Center Groningen, Groningen, The Netherlands; 3 Department of Paediatric Intensive Care, Amsterdam UMC, Amsterdam, The Netherlands; 4 Critical Care, Anaesthesiology, Peri-Operative Medicine & Emergency Medicine, The University of Groningen, Groningen, The Netherlands

**Keywords:** HFOV, Pediatric, Staircase lung volume optimization maneuver, Respiratory inductance plethysmography

## Abstract

**Background:**

Titration of the continuous distending pressure during a staircase incremental–decremental pressure lung volume optimization maneuver in children on high-frequency oscillatory ventilation is traditionally driven by oxygenation and hemodynamic responses, although validity of these metrics has not been confirmed.

**Methods:**

Respiratory inductance plethysmography values were used construct pressure–volume loops during the lung volume optimization maneuver. The maneuver outcome was evaluated by three independent investigators and labeled positive if there was an increase in respiratory inductance plethysmography values at the end of the incremental phase. Metrics for oxygenation (SpO_2_, FiO_2_), proximal pressure amplitude, tidal volume and transcutaneous measured pCO_2_ (p_tc_CO_2_) obtained during the incremental phase were compared between outcome maneuvers labeled positive and negative to calculate sensitivity, specificity, and the area under the receiver operating characteristic curve. Ventilation efficacy was assessed during and after the maneuver by measuring arterial pH and PaCO_2_. Hemodynamic responses during and after the maneuver were quantified by analyzing heart rate, mean arterial blood pressure and arterial lactate.

**Results:**

41/54 patients (75.9%) had a positive maneuver albeit that changes in respiratory inductance plethysmography values were very heterogeneous. During the incremental phase of the maneuver, metrics for oxygenation and tidal volume showed good sensitivity (> 80%) but poor sensitivity. The sensitivity of the SpO_2_/FiO_2_ ratio increased to 92.7% one hour after the maneuver. The proximal pressure amplitude showed poor sensitivity during the maneuver, whereas tidal volume showed good sensitivity but poor specificity. PaCO_2_ decreased and pH increased in patients with a positive and negative maneuver outcome. No new barotrauma or hemodynamic instability (increase in age-adjusted heart rate, decrease in age-adjusted mean arterial blood pressure or lactate > 2.0 mmol/L) occurred as a result of the maneuver.

**Conclusions:**

Absence of improvements in oxygenation during a lung volume optimization maneuver did not indicate that there were no increases in lung volume quantified using respiratory inductance plethysmography. Increases in SpO_2_/FiO_2_ one hour after the maneuver may suggest ongoing lung volume recruitment. Ventilation was not impaired and there was no new barotrauma or hemodynamic instability. The heterogeneous responses in lung volume changes underscore the need for monitoring tools during high-frequency oscillatory ventilation.

## Background

Preventing ventilator-induced lung injury (VILI) is one of the many challenges when ventilating critically ill children, especially since the exact underlying mechanisms and consequences in pediatrics are far from understood [1]. Little pediatric evidence supports best ventilation practices that can be applied to attenuate the detrimental effects of two key factors of VILI, being atelectrauma [the repetitive opening and closure of alveoli] and volutrauma [the delivery of large tidal volume (*V*_*t*_) in relation to the amount of inflatable lung volume]. From a theoretical perspective, high-frequency oscillatory ventilation (HFOV) is an ideal mode for lung-protective ventilation given its delivery of very small stroke volumes and pressure changes at the alveolar level while preserving end-expiratory lung volume (EELV).

Pediatric critical care practitioners have embraced HFOV for the management of acute respiratory failure, despite the outcome of the only pediatric randomized clinical trial (RCT) failing to show benefit on patient outcome [2]. Also, continued use became controversial following the publication of two clinical trials in adult ARDS in 2013 [3, 4]. Whereas patient outcomes including all-cause mortality were similar in the Oscillation for ARDS (OSCAR) trial, the High Frequency Oscillation for Early ARDS (OSCILLATE) trial was stopped prematurely because of increased mortality among those randomized to HFOV [3, 4]. However, the question is whether the outcomes of these studies confirmed that HFOV was not beneficial or even harmful, or if it was a matter of how the oscillator was used that determined patient outcomes [5, 6]. For example, most adult and pediatric observational studies not reported using lung volume optimization maneuvers (LVOM). Nonetheless, experimental work underscored the importance of recruiting lung volume when transitioning to HFOV because not only oxygenation improves with more recruited lung volume, but also to prevent collapsed, atelectatic lung units from becoming exposed to larger, potentially more injurious pressure swings [7, 8]. However, the best lung volume optimization maneuver has yet to be identified both in children and adults [9].

One approach to a lung volume optimization maneuver is the staircase incremental–decremental pressure titration [10–12]. Since it has been proposed that oxygenation improves linearly with increases in lung volume during HFOV, the transcutaneous measured oxygen saturation (SpO_2_) in relation to the delivered oxygen fraction (FiO_2_) drives the pressure titration. In addition, the proximal pressure amplitude is monitored during the maneuver because it may be used as a marker of respiratory system compliance. Lastly, a decrease in blood pressure may be interpreted as sign of lung overdistension [9]. The unanswered question is how reliable these variables are to identify the onset of lung volume recruitment and lung overdistension [13, 14]. Recently, we reported that changes in the cross-sectional area of the chest wall and the abdominal compartment measured with respiratory inductance plethysmography (RIP) may act as indicator for lung volume changes during a lung volume optimization maneuver [15]. The present efficacy and safety study builds on our pilot work. We sought to explore how changes in metrics for oxygenation (SpO_2_ and FiO_2_), proximal pressure amplitude and tidal volume reflected changes in lung volume during and after the lung volume optimization maneuver (efficacy). Next, we sought to study the effect of the lung volume optimization maneuver on CO_2_ clearance, occurrence of hemodynamic instability and development of new barotrauma (safety).

## Methods

### Study population

We collected data during the lung volume optimization maneuver in children after the indication for HFOV was set by the attending physician in agreement with the clinical guideline [10]. Children were enrolled after written informed consent from either parents or legal caretakers if they had acute onset of lung disease and were on neuromuscular paralysis. Children with a gestational age < 40 weeks, with congenital or acquired paralysis of the diaphragm, uncorrected congenital heart disorder, severe pulmonary hypertension or open thorax or abdomen after surgery and those with status asthmaticus were excluded. The Institutional Review Board of the hospital approved the study. PARDS was defined as previously described [16].

### HFOV lung volume optimization strategy

Initial oscillator settings irrespective of age or bodyweight included frequency 10–12 Hz, continuous distending pressure 3–5 cm H_2_O above mean airway pressure (mPaw) on conventional mechanical ventilation (CMV), power setting targeting 70–90 cmH_2_O proximal pressure amplitude, inspiratory time 33% and bias flow 20–40 L/min. Immediately after transitioning to HFOV using the SensorMedics 3100A/B oscillator (SensorMedics, Yorba Linda, CA), a quasi-static lung volume optimization maneuver was performed by an individualized staircase incremental–decremental pressure titration, allowing to map the pressure–volume (PV) relationship. Briefly, the inflation limb was mapped by increasing pressure 2 cmH_2_O every 3–5 min with ongoing oscillations while simultaneously monitoring the SpO_2_ and mean arterial blood pressure, identifying the onset of lung recruitment (i.e., increase in SpO_2_) until no further improvement in oxygenation and/or sudden decrease in mean arterial blood pressure during two consecutive pressure increments. Subsequently, the pressure was stepwise decreased every 3–5 min by 2 cm H_2_O until the SpO_2_ decreased again indicating lung derecruitment or when other clinical situations gave rise to stop decreasing the pressure. Frequency and power remained constant, so that the proximal pressure amplitude could reflect changes in respiratory system compliance. Only in patients with severe increasing hypercapnia resulting in acidosis (pH < 7.25), frequency was lowered during the maneuver [10].

### Measurements and data acquisition

Mean arterial blood pressure (Edwards Lifesciences, Irvine, CA, USA), SpO_2_ (Masimo Corporation, Irvine, CA, USA), transcutaneous CO_2_ (Radiometer, Brønshøj, Denmark) and tidal volume were continuously measured during the maneuver. Continuous distending pressure and proximal pressure amplitude were read from the oscillator. Arterial blood samples were taken to measure PaCO_2_, pH and lactate before and 1 h after the maneuver (Radiometer, Brønshøj, Denmark). Tidal volume was measured using an inline hot wire flow sensor (Acutronic, Hirzel, Switzerland). Respiratory inductance plethysmography signals were sampled at 200 Hz using two elastic bands (Nox Medical, Reykjavik, Iceland) connected to the Bicore II™ (Vyaire, Yorba Linda, Ca, USA) and stored for offline analysis. Uncalibrated signals were derived from the chest and abdominal respiratory inductance plethysmography signals. Data acquisition was done using a custom-built Polybench software module (Applied Biosignals, Weener, Germany).

### Data analysis

All data analyses were done offline. First, to study how changes in metrics for oxygenation (SpO_2_ and FiO_2_), proximal pressure amplitude and tidal volume reflected changes in lung volume during the lung volume optimization maneuver, we normalized the continuous distending pressure by setting the pressure at which the maneuver started (initial and lowest pressure) at 0% and the last (highest) pressure during the incremental phase of the maneuver at 100%. We calculated mean respiratory inductance plethysmography signals (chest, abdomen and sum) from a 30-s artifact-free period just before the next increase in pressure allowing for lung volume equilibration, using a custom-built Polybench software module (Applied Biosignals, Weener, Germany). These values were then plotted against the normalized pressure to map the pressure–volume relationship according to the four-parameter logistic Venegas equation provided that enough data points were available to plot [17]. The lung volume optimization maneuver was labeled responsive if there was an increase in respiratory inductance plethysmography signal during the incremental phase as we previously have done [15]. Then, we plotted tidal volume against pressure using the four-parameter logistic Venegas equation [17]. SpO_2_, SpO_2_/FiO_2_, proximal pressure amplitude and the transcutaneous pCO_2_ were also plotted against pressure. Subsequently, three independent pediatric clinician-researchers (PdJ, RB, MK) classified these plots positive (i.e., improvement in SpO_2_ and/or SpO_2_/FiO_2_ ratio, tidal volume, decrease in proximal pressure amplitude) or negative by visual inspection. Intraclass correlation coefficient (ICC) Fleiss kappa was calculated to evaluate the agreement between investigators.

Next, we wanted to study the time course of the SpO_2_/FiO_2_ ratio, proximal pressure amplitude, tidal volume, PaCO_2_, pH and changes in mean arterial blood pressure and arterial lactate by comparing four time points: just before transitioning to high-frequency oscillatory ventilation (T0), at the end of the incremental phase when there was the highest applied pressure (T1), at the last applied pressure during the decremental phase (T2) and 1 h (T3) after the maneuver. For analytical purposes, we compared the number of patients with arterial blood lactate > 2 mmol/L and mean arterial blood pressure lower than the age-adjusted p10 or ≥ p90.

### Statistical analyses

The primary endpoint was sensitivity and specificity of metrics for oxygenation, proximal pressure amplitude and tidal volume to reflect lung volume changes during the maneuver. Secondary endpoints included the time course of the SpO_2_/FiO_2_ ratio, proximal pressure amplitude and tidal volume from baseline to 1 h after the maneuver, effects of the maneuver on ventilation parameters (PaCO_2_ and pH), occurrence of hemodynamic instability and development of new barotrauma. Continuous data are presented as median and 25–75 interquartile range (IQR) and absolute numbers as percentage of total. Continuous paired data were tested using the Wilcoxon signed rank test and comparisons between groups with the Mann–Whitney U test. Dichotomous variables were tested with the χ^2^ test or the Fisher exact test if values were < 5. Sensitivity and specificity were calculated for all plots and the SpO_2_/FiO_2_ ratio at T2 and T3. *P* values less than 0.05 were accepted as statistically significant.

## Results

### Patient characteristics

Fifty-two patients completed the maneuver, with two patients having two separate HFOV runs in different ventilation periods (Table 1). Primary admission diagnosis was acute respiratory failure in 48 patients, tracheal rupture in one and sepsis in the remaining five. All but ten patients met PARDS criteria. Baseline SpO_2_/FiO_2_ ratio of the whole cohort was 190 (121–243). Median CMV settings prior to HFOV were peak inspiratory pressure (PIP) 31 cmH_2_O (30–34), PEEP 8 cmH_2_O (6–8) and FiO_2_ 0.5 (0.4–0.76).

**Table 1:** Patient characteristics

Number of patients	54
Male/female (%)	56/44
Age (months)	3.3 (1.7–12.3)
< 12 months (%)	75.9
12–24 months	3.7
> 24 months	20.4
Weight (kg)	6 (4.5–9.1)
PRISM II (24 h) score	14 (11–17)
Pulmonary admission diagnosis (%)	87.1
PARDS (%)	81.5
Mild (%)	15.9
Moderate (%)	59.1
Severe (%)	25.0
PIP (cmH_2_O)	31 (30–34)
PEEP (cm H_2_O)	8 (6–8)
FiO_2_	0.5 (0.4–0.76)
SpO_2_/FiO_2_ ratio on CMV	190 (121–243)
OI on CMV	10.2 (8.3–15.7)
pH	7.3 (7.23–7.35)
pCO_2_ on CMV (mmHg)	57 (52–64)
Duration of MV (days)	6.6 (4.9–10.4)
Extra-corporeal membrane support (%)	1.9
Mortality (%)	1.9

*PRISM* pediatric risk of mortality, *PARDS* pediatric acute respiratory distress syndrome, *PIP* peak inspiratory pressure, *PEEP* positive end-expiratory pressure, *OI* oxygenation index, *MV* mechanical ventilation

The median highest pressure at the end of the incremental phase of the maneuver after a median number of incremental steps of 8 (7–9) was 34 (32–36) cmH_2_O (Additional file 1: Table S1). The maneuver was labeled responsive in 41 patients (75.9%) by the three independent reviewers (Fleiss’ kappa 0.967). Pooled data plotted according to Venegas did not show a clear overall lower and upper inflection point (Additional file 2: Figure S1).

### *SpO*_*2*_*/FiO*_*2*_* ratio, proximal pressure amplitude and tidal volume as predictor for lung volume changes during the maneuver*

**Fig. 1:**
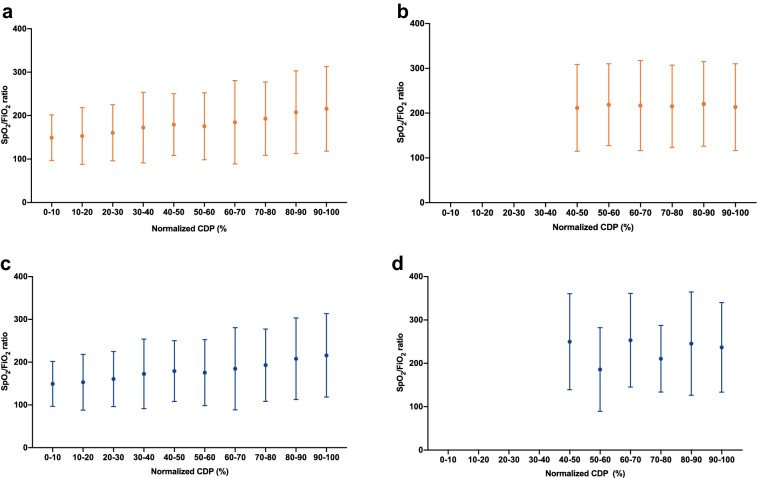
Pooled SpO_2_/FiO_2_ ratio for a given continuous distending pressure (CDP) normalized from 0 to 100% during the lung volume optimization maneuver incremental phase stratified by outcome, i.e., responsive (panel A and B, N = 41) or unresponsive (panel C and D, N = 13). Data are presented as median and 25–75 interquartile range

Table 2 summarizes the number of plots labeled positive or negative by visual inspection stratified by lung volume optimization maneuver outcome (i.e., responsive, or unresponsive). Metrics for oxygenation (i.e., the SpO_2_–pressure and/or SpO_2_/FiO_2_–pressure plot showed a high sensitivity of 87.8%, but the sensitivity was 53.8% and there was disagreement among the three independent reviewers (Fleiss’ kappa 0.431). Heterogeneity in the pooled SpO_2_/FiO_2_ ratio for a given normalized pressure during the incremental (Fig. 1a, b) and decremental phase (Fig. 1c, d) of the maneuver stratified by outcome reflected the mediocre negative predictive value of 58.3%. Proximal pressure amplitude (negative predictive value 25.6%) or tidal volume (negative predictive value 20.0%) showed even lower negative predictive values.

**Table 2:** Classification results from plots of metrics for oxygenation, proximal pressure amplitude (Δ*P*_proximal_) and tidal volume (*V*_*t*_) plotted against the continuous distending pressure (CDP) with corresponding sensitivity, specificity, positive and negative predictive value calculated using the respiratory inductance plethysmography–CDP plots as reference, and Fleiss’ Kappa

Variable	Changes observed	LVOM outcome		Sensitivity	Specificity	Positive predictive value	Negative predictive value	ROC AUC (95% CI)	Fleiss kappa
		Responsive	Unresponsive						
During lung volume optimization maneuvre									
Oxygenation–CDP	Increase, suggestive for lung volume recruitment	36/41	6/13	87.8	53.8	85.7%	58.3%	0.71 (0.53–0.89)	0.431
	No change or decrease	5/41	7/13						
Δ*P*_proximal_–CDP	Decrease, suggestive for increased compliance	12/41	3/13	29.3	76.9	80.0%	25.6%	0.53 (0.35–0.71)	0.782
	No change or increase	29/41	10/13						
*V*_*t*_–CDP curve	Increase, suggestive for lung volume recruitment	31/35	10/11	88.6	9.1	75.6%	20.0%	0.49 (0.29–0.68)	0.866
	No change or decrease	4/35	1/11						
After lung volume optimization maneuvre									
SpO_2_/FiO_2_ ratio at the end of the maneuvre	Increase ≥ 20%	22/41	5/13	53.7	61.5	81.4%	29.6%	0.58 (0.40–0.75)	N/A
	Decrease or increase < 20%	19/41	8/13						
SpO_2_/FiO_2_ ratio 1 h after the maneuvre	Increase ≥ 20%	38/41	3/13	92.7	76.9	92.6%	76.9%	0.85 (0.70–0.99)	N/A
	Decrease or increase < 20%	3/41	10/13						

The lower panel summarizes changes in SpO_2_/FiO_2_ ratio at the last applied CDP during the decremental phase (T2) and one hour after (T3) after the lung volume optimization maneuver (LVOM). Data are depicted as median (25–75 interquartile range) or absolute number. ROC AUC receiver operating characteristic area under the curve; 95% confidence interval

### *Time course of SpO*_*2*_*/FiO*_*2*_* ratio, proximal pressure amplitude and tidal volume*

Except for pressure at the end of the incremental phase or at the end of the lung volume optimization maneuver, oscillator settings remained constant over time, (Additional file 1: Table S1). For the whole cohort the median SpO_2_/FiO_2_ ratio increased from 190 (121–243) before the maneuver to 240 (167–324) 1 h after the maneuver (*p* < 0.001). Significant improvements in SpO_2_/FiO_2_ ratio were observed in responsive and unresponsive lung volume optimization maneuvers (Table 3). Sensitivity, specificity, and positive and negative predictive value of SpO_2_/FiO_2_ were better one hour after the lung volume optimization maneuver (Table 2). Tidal volume, but not the proximal pressure amplitude did not significantly change over time, irrespective of lung volume optimization maneuver outcome (Table 3). Strong heterogeneity in tidal volume changes during the incremental and decremental phase of the lung volume optimization maneuver was observed (Additional file 3: Figure S2).

**Table 3:** Summary of changes in SpO2/FiO2, pCO2, pH, ΔPproximal and tidal volume (*V*_*t*_), assessed just before the lung volume optimization maneuver (LVOM) when the patients were on conventional mechanical ventilation (CMV) [T0], at the last applied CDP during the incremental (T1) and decremental phase (T2), and one hour after the LVOM [T3]

	Lung volume optimization maneuver outcome							
	Responsive (*N* = 41)				Unresponsive (*N* = 13)			
	T0	T1	T2	T3	T0	T1	T2	T3
SpO_2_/FiO_2_	186 (121–243)	190 (141–315)	188 (139–319)	240 (174–327)	218 (151–242)	243 (145–299)	238 (144–290)	242 (154–265)
Proximal pressure amplitude	78 (72–85)	82 (75–86)	82 (77–86)	76 (73–82)	77 (69–84)	81 (78–89)	80 (72–83)	75 (72–81)
Tidal volume	0.9 (0.7–1.2)	1.6 (1.3–1.8)	1.6 (1.3–1.7)		1 (.7–1.2)	1.6 (0.9–2.2)	1.6 (1.3–1.8)	

Data are depicted as median (25–75 interquartile range) or absolute number

### Safety of the lung volume optimization maneuver

The overall median arterial pH improved from 7.30 (7.23–7.35) before the maneuver to 7.38 (7.32–7.45, *p* < 0.001) 1 h after the maneuver. We also observed improvements in pCO_2_ from (58 (52–64) before the maneuver to 46 (36–53) mmHg, *p* < 0.001) 1 h after the maneuver. Significant changes were observed irrespective of lung volume optimization maneuver outcome (Additional file 4: Table S2). No new barotrauma or hemodynamic instability occurred (Additional file 5: Table S3). Fifteen (27.8%) patients received a fluid bolus during the maneuver.

## Discussion

This study is one of the first studies to characterize physiologic responses to a lung volume optimization maneuver during pediatric high-frequency oscillatory ventilation. We confirmed our previous preliminary observations that clinical variables used at the bedside to identify changes in lung volume correlated poorly with lung volume changes measured using respiratory inductance plethysmography [15]. We also report that an individual staircase lung volume optimization maneuver was safe and did not negatively impact CO_2_ elimination.

Optimization of lung volume after switching to HFOV is necessary to minimize lung injury and to allow for a better dampening of the pressure swings as well as oscillation on the deflation limb of the pressure–volume loop where there is less continuous alveolar recruitment and derecruitment [7, 18–20]. Despite this physiologic rationale, there is very little data regarding the optimal lung volume optimization maneuver except for one neonatal lamb model study comparing a staircase CDP titration with (repeated) sustained inflations (SI) [11]. A stepwise CDP increase produced the greatest increase in lung volume and resolution of atelectasis compared with a 20-s SI (either once or repeated 6 times) or a standard approach (i.e., randomly setting the CDP) [11]. SIs have been studied in relevant animal models, but no comparisons were made with other lung volume optimization maneuvers [21, 22]. By using a staircase incremental CDP titration, we observed a highly variable response to titration among the studied patients. Many studied patients showed a lung volume increase and individual lower (LIP) and upper inflection points (UIP) identified, but there was no clear overall LIP or UIP point during the inflation phase of the lung volume optimization maneuver (Fig. 1). Even though our study was not designed to identify the best lung volume optimization maneuver, the heterogeneity that we observed may put the lung volume optimization maneuver used in the OSCILLATE trial in a different perspective. In that trial, a SI of 40 cmH_2_O for 40 s was used, but it can be argued that such a standardized, non-individualized lung volume optimization maneuver may have led to either lung overdistension or poorly recruited alveoli contributing to poor patient outcome [3, 5, 6]. We also observed heterogeneity during the deflation phase (Additional file 2: Figure S1), questioning if routinely reducing the CDP to a certain level to find the sweet spot of ventilation as was done in the OSCILLATE trial is justified. Our data underscore the need for further studies into the most optimal lung volume optimization maneuver.

The uncertainty about lung volume optimization maneuver is further fueled by the fact that it is unclear how to bedside monitor changes in lung volume. Traditionally, clinical parameters such as SpO_2_, OI and PaO_2_/FiO_2_ are used to monitor changes in lung volume [13, 14]. Preliminary experimental and clinical work in neonates supported the possibility of monitoring lung volume changes during HFOV with respiratory inductance plethysmography [12, 23–25]. Respiratory inductance plethysmography is a validated, noninvasive respiratory monitoring technique used as proxy for changes in lung volume by quantifying changes in the cross-sectional area of the chest wall and the abdominal compartment [26, 27]. Previously, we also reported that RIP may be of additional value during pediatric HFOV [15]. In our present study, we found that oxygenation improved not only in responsive, but also unresponsive lung volume optimization maneuvers, challenging if oxygenation is a good bedside marker for lung volume changes as also has been observed during CMV [28, 29]. It has also been proposed that a decrease in blood pressure might be perceived as sign of lung overdistension and may thus be used as a parameter guiding the lung volume optimization maneuver. In line with our previously reported pilot data, we very infrequently observed this phenomenon [15]. It can also be argued that we did not fully recruit the lung, hence explaining our failure to observe a decrease in blood pressure and even suggesting potential for delivering a higher CDP during the lung volume optimization maneuver. However, it has never been studied if a heterogeneous diseased lung should be recruited up to the point of a decrease in blood pressure, as the inherent risk is that some areas may be already overdistended at even low airway pressures [30, 31]*.*

Traditionally, HFOV decouples oxygenation and ventilation. However, we observed an improvement in pCO_2_ and pH one hour post lung volume optimization maneuver, irrespective of the response status or changes in F. Also, changes in tidal volume were seen in both responsive and unresponsive lung volume optimization maneuvers. Our findings may be interpreted as that HFOV may exert also positive effects not only on oxygenation, but also ventilation.

Our study may have two clinical implications. First, we observed that oxygenation improved further after the lung volume optimization maneuver, yielding a better sensitivity and specificity compared with oxygenation immediately post lung volume optimization maneuver. The significant decrease in proximal pressure amplitude without changes in oscillatory power in responsive lung volume optimization maneuvers suggests an ongoing improvement in lung compliance post maneuver. Hence, we postulate that the clinical assessment of whether the lung volume optimization maneuver was successful should be done in the hours following the maneuver. Second, the staircase lung volume optimization maneuver was safe in both responsive and unresponsive lung volume optimization maneuvers as there was no significant hypercapnia despite using a high frequency nor did it lead to hemodynamic impairment or new barotrauma.

The strength of this study is that it is the first clinical pediatric study characterizing the lung volume optimization maneuver during HFOV coming from a high-volume HFOV unit, reporting limited applicability of traditionally used clinical variables and safety and feasibility of a staircase, personalized lung volume optimization maneuver. However, there are also several limitations that need to be discussed. First, as our study was a single-center study, this may limit the generalizability of our findings. The study cohort was an inhomogeneous population with different causes and stages of the lung disease and different degrees of PARDS. However, we are confident that this does not reduce the validity of the data. Second, we measured changes in respiratory inductance plethysmography as proxy of lung volume changes. Validation against any other lung imaging technique such as CT or electrical impedance tomography (EIT) may be necessary because the respiratory inductance plethysmography signal does not distinguish between global changes in lung and thoracic blood volume [23, 27]. Nonetheless, respiratory inductance plethysmography has been shown to estimate mean lung volume changes during HFOV and the lack of cardiovascular instability observed in our study suggests that most (if not all) of the respiratory inductance plethysmography changes were due to changes in thoracic gas volume [23, 32, 33]. Lastly, we did not a priori define how much the respiratory inductance plethysmography signal needed to improve to be labeled as responsive lung volume optimization maneuver when mapping these against pressure. Again, this requires comparison with other measures of lung volume change such as EIT or CT.

## Conclusions

Clinically used bedside variables commonly used during a lung volume optimization maneuver with pediatric HFOV correlate poorly with lung volumes changes measured using respiratory inductance plethysmography. A personalized staircase lung volume optimization maneuver does not cause hemodynamic impairment or negatively impacts CO_2_ elimination. Further studies are necessary to compare the maneuver with other maneuvers and study the effect of a respiratory inductance plethysmography-guided maneuver on patient outcome.

## Supplementary information

**Additional file 1:** **Table S1.** Oscillator settings at start of the lung volume optimization maneuver, at the end of the incremental phase, at the end of the decremental phase and one hour after completion of the maneuver.

**Additional file 2:** **Figure S1.** Pooled changes in respiratory inductance plethysmography (RIP) measured during the lung volume optimization maneuver for a given continuous distending pressure (CDP) (normalized from 0 to 100%).

**Additional file 3:** **Figure S2.** Pooled changes in tidal volume (Vt) measured during the lung volume optimization maneuver for a given continuous distending pressure (CDP) (normalized from 0 to 100%) fitted according to Venegas for the incremental phase and decremental phase.

**Additional file 4:** **Table S2.** Summary of changes in PaCO2 and pH.

**Additional file 5:** **Table S3.** Comparison between the number of patients with hemodynamic instability and new barotrauma before and after the lung volume optimization maneuver.

## Data Availability

The datasets used and analyzed during the current study are available from the corresponding author on reasonable request.

## Abbreviations

ARDS: Acute respiratory distress syndrome
CDP: Continuous distending pressure
CDP_hyperinflation_: CDP point representing the onset of hyperinflation
CDP_recruitment_: CDP point representing the onset of lung recruitment
CMV: Conventional mechanical ventilation
EELV: End-expiratory lung volume
EIT: Electrical impedance tomography
F: Frequency
FiO2: Delivered oxygen fraction
HFOV: High-frequency oscillatory ventilation
ICC: Intraclass correlation coefficient
IQR: Interquartile range
LIP: Lower inflection point
LVOM: Lung volume optimization maneuver
mABP: Mean arterial blood pressure
mPaw: Mean airway pressure
MV: Mechanical ventilation
OI: Oxygenation index
PARDS: Pediatric acute respiratory distress syndrome
PEEP: Positive end-expiratory pressure
PIP: Peak inspiratory pressure
Δ*P*_proximal_: Proximal pressure amplitude
PRISM: Pediatric risk of mortality
p_tc_CO_2_: Transcutaneous measured pCO_2_
P–V: Pressure–volume (PV)
RCT: Randomized clinical trial
RIP: Respiratory inductance plethysmography
SI: Sustained inflations
SpO_2_: Transcutaneous measured oxygen saturation
T0: Time point just before transitioning to HFOV
T1: Time point at the highest applied CDP at the end of the incremental phase
T2: Time point at the last applied CDP during the decremental phase
T3: Time point 1 h after the LVOM
UIP: Upper inflection point
VILI: Ventilator-induced lung injury
*V*_*t*_: Tidal volume
