# Supplementary material for: Physiologic responses to a staircase lung volume optimization maneuver in pediatric high-frequency oscillatory ventilation
Source: Ann Intensive Care. 2020 Nov 18;10:153. doi: 10.1186/s13613-020-00771-8 (PMC7672171; doi:10.1186/s13613-020-00771-8)
Supplement: Supplementary file 1 — Additional file 1: Table S1. Oscillator settings at start of the lung volume optimization maneuver, at the end of the incremental phase, at the end of the decremental phase and one hour after completion of the maneuver. [file 13613_2020_771_MOESM1_ESM.docx]

**Additional file 1 – Table S1**

| **Time point** | **Lung volume optimization maneuver outcome** | **Oscillator Settings** | | | **No. of CDP steps** |
| --- | --- | --- | --- | --- | --- |
|  |  | **CDP (cmH_2_O)** | **Frequency (Hz)** | **Proximal pressure amplitude (cmH_2_O)** |  |
| Before the maneuver | All (N = 54) | 20 (18-22) | 12 (12-12) | 78 (71-85) | N/A |
|  | Responsive (N = 41) | 20 (18-22) | 12 (12-12) | 79 (72-85) | N/A |
|  | Unresponsive (N = 13) | 22 (19-22) | 12 (12-12) | 77 (69-82) | N/A |
| At the end of the incremental phase | All (N = 54) | 34 (32-36) | 12 (12-12) | 82 (77-86) | 8 (7-9) ‡ |
|  | Responsive (N = 41) | 35 (33-37)* | 12 (12-12) | 82 (76-86) | 8 (7-10) ‡ |
|  | Unresponsive (N = 13) | 33 (29-35)* | 12 (10-12) | 81(78-89) | 7 (6-8) ‡ |
| At the end of the maneuver | All (N = 54) | 26 (24-28) | 12 (10-12) | 81 (77-85) | 6 (4-7) † |
|  | Responsive (N = 41) | 26 (24-28)* | 12 (12-12) | 82 (77-86) | 6 (4-7) † |
|  | Unresponsive (N = 13) | 24 (21-27)* | 12 (9-12) | 80 (72-83) | 6 (4-7) † |
| 1 hour after the maneuver | All (N = 54) | 25 (23-27) | 12 (9-12) | 76 (72-82) | N/A |
|  | Responsive (N = 41) | 26 (24-28) | 12 (10-12) | 76 (73-82) | N/A |
|  | Unresponsive (N = 13) | 24 (22-27) | 12 (9-12) | 75 (72-81) | N/A |

Oscillator settings at start of the lung volume optimization maneuver, at the end of the incremental phase, at the end of the decremental phase and one hour after completion of the maneuver. Number of steps indicates the number of pressure increments during the inflation (denoted by ‡) and deflation (denoted by †) phase. Data are depicted as median (25 – 75 interquartile range). CDP continuous distending pressure; N/A not applicable. * denotes p < 0.05.
