# Supplementary material for: Physiologic responses to a staircase lung volume optimization maneuver in pediatric high-frequency oscillatory ventilation
Source: Ann Intensive Care. 2020 Nov 18;10:153. doi: 10.1186/s13613-020-00771-8 (PMC7672171; doi:10.1186/s13613-020-00771-8)
Supplement: Supplementary file 2 — Additional file 2: Figure S1. Pooled changes in respiratory inductance plethysmography (RIP) measured during the lung volume optimization maneuver for a given continuous distending pressure (CDP) (normalized from 0 to 100%). [file 13613_2020_771_MOESM2_ESM.docx]

**Additional file 2 – Figure S1**


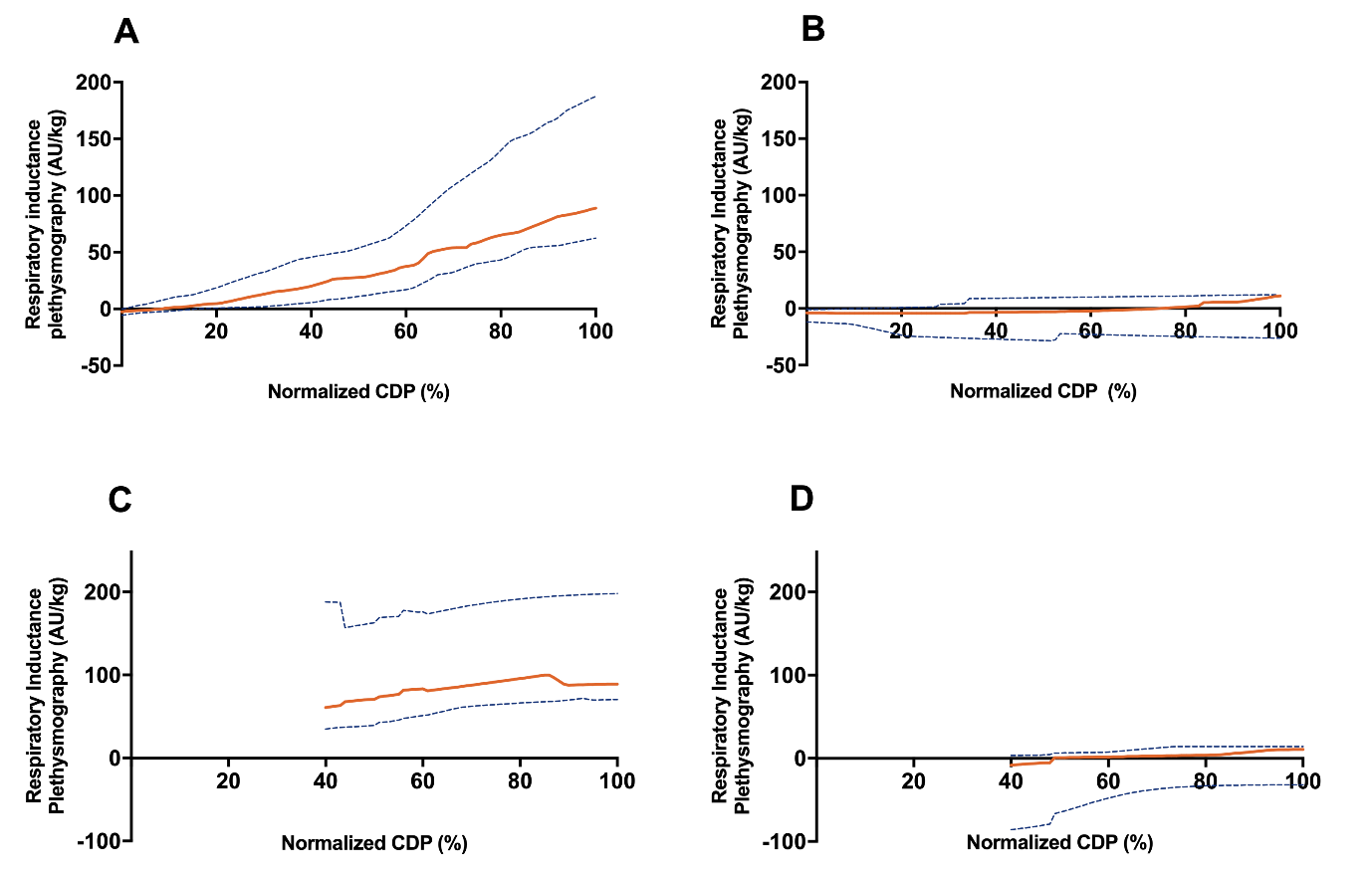


Pooled changes in respiratory inductance plethysmography (RIP) (expressed in arbitrary units per kg actual bodyweight) measured during the lung volume optimization maneuver for a given continuous distending pressure (CDP) (normalized from 0 to 100%) fitted according to Venegas for the incremental phase (panel A [responsive] and B [unresponsive]) and for the decremental phase (panel C [responsive] and D [unresponsive]) if more than three decremental pressure steps were available, stratified by maneuver outcome (response [N = 41] or unresponsive [N = 13]) defined by visual inspection. Both during the incremental and decremental phase, a clear lower and upper inflection could not be identified. Data are presented as median (orange line) and 25-75 interquartile range (dotted blue lines).
