# Supplementary material for: Physiologic responses to a staircase lung volume optimization maneuver in pediatric high-frequency oscillatory ventilation
Source: Ann Intensive Care. 2020 Nov 18;10:153. doi: 10.1186/s13613-020-00771-8 (PMC7672171; doi:10.1186/s13613-020-00771-8)
Supplement: Supplementary file 3 — Additional file 3: Figure S2. Pooled changes in tidal volume (Vt) measured during the lung volume optimization maneuver for a given continuous distending pressure (CDP) (normalized from 0 to 100%) fitted according to Venegas for the incremental phase and decremental phase. [file 13613_2020_771_MOESM3_ESM.docx]

**Additional file 3 – Figure S2**


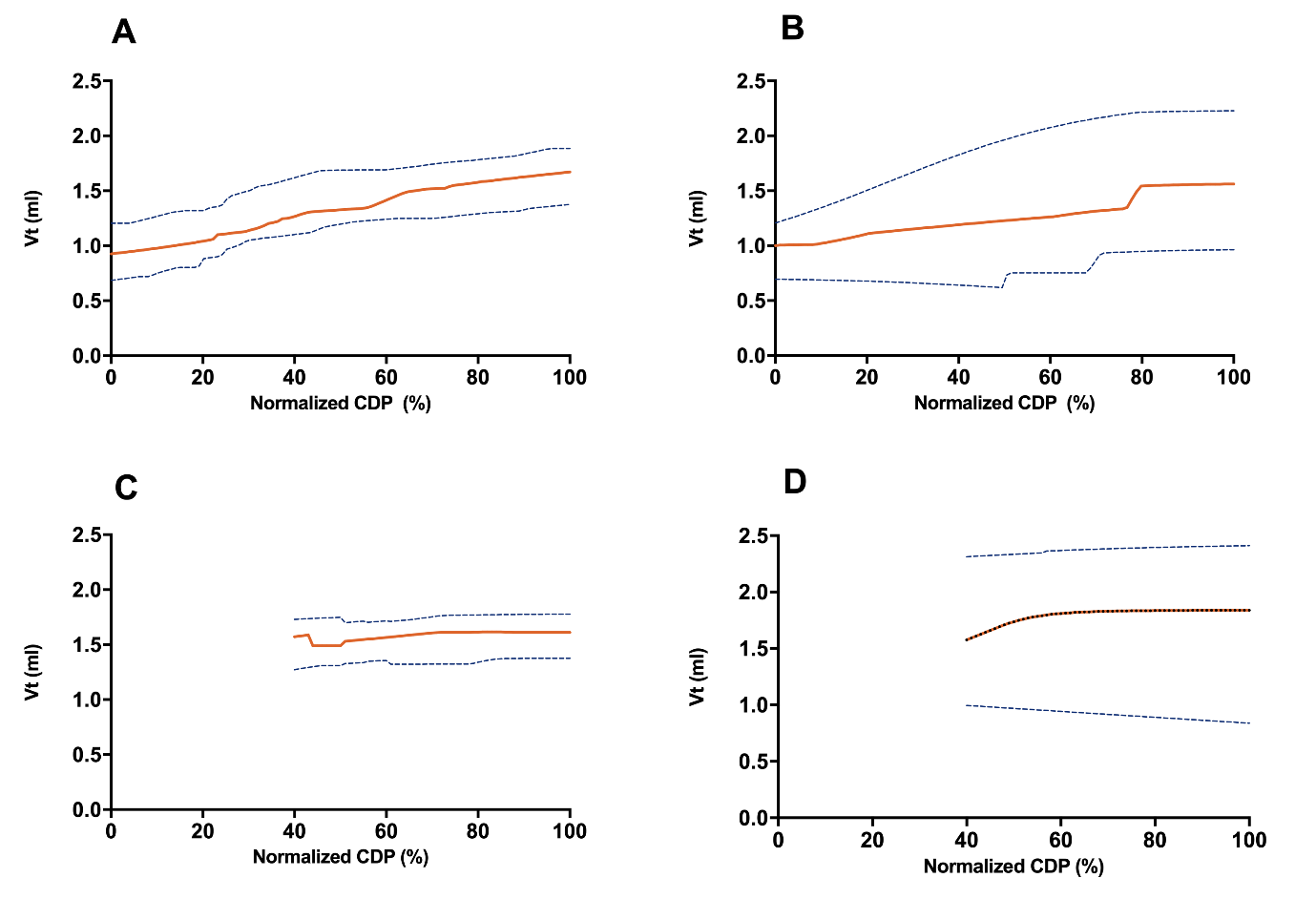


Pooled changes in tidal volume (Vt) measured during the lung volume optimization maneuver for a given continuous distending pressure (CDP) (normalized from 0 to 100%) fitted according to Venegas for the incremental phase (panel A [responsive] and B [unresponsive]) and for the decremental phase (panel C [responsive] and D [unresponsive]) if more than three decremental pressure steps were available, stratified by maneuver outcome (response [N = 41] or unresponsive [N = 13]) defined by visual inspection. Both during the incremental and decremental phase, a clear lower and upper inflection could not be identified. Data are presented as median (orange line) and 25-75 interquartile range (dotted blue lines).
