# Supplementary material for: Physiologic responses to a staircase lung volume optimization maneuver in pediatric high-frequency oscillatory ventilation
Source: Ann Intensive Care. 2020 Nov 18;10:153. doi: 10.1186/s13613-020-00771-8 (PMC7672171; doi:10.1186/s13613-020-00771-8)
Supplement: Supplementary file 4 — Additional file 4: Table S2. Summary of changes in PaCO2 and pH. [file 13613_2020_771_MOESM4_ESM.docx]

**Additional file 4 – Table S3**

|  | Lung volume optimization maneuver outcome | | | |
| --- | --- | --- | --- | --- |
|  | Responsive (N = 41) | | Unresponsive (N = 13) | |
|  | Before maneuver | 1 hour after maneuver | Before maneuver | 1 hour after maneuver |
| pCO_2_ (mmHg) | 56  (51-64) | 41*  (35-51) | 62  (54-65) | 48*  (46-58) |
| pH | 7.31  (7.21-7.36) | 7.39*  (7.32-7.46) | 7.28  (7.25-7.34) | 7.37*  (7.31-7.41) |

Summary of changes in PaCO_2_ and pH. Data are depicted as median (25 – 75 interquartile range). * denotes *p* < 0.05
