# Supplementary material for: Physiologic responses to a staircase lung volume optimization maneuver in pediatric high-frequency oscillatory ventilation
Source: Ann Intensive Care. 2020 Nov 18;10:153. doi: 10.1186/s13613-020-00771-8 (PMC7672171; doi:10.1186/s13613-020-00771-8)
Supplement: Supplementary file 5 — Additional file 5: Table S3. Comparison between the number of patients with hemodynamic instability and new barotrauma before and after the lung volume optimization maneuver. [file 13613_2020_771_MOESM5_ESM.docx]

**Additional file 5 – Table S4**

|  | Lung volume optimization maneuver outcome | | | |
| --- | --- | --- | --- | --- |
|  | Responsive | | Unresponsive | |
|  | Before maneuver | 1 hour after maneuver | Before maneuver | 1 hour after maneuver |
| Bloodpressure < p10 | 4 | 2 | 1 | 1 |
| Bloodpressure ≥ p90 | 4 | 2 | 2 | 1 |
| Lactate > 2 mmol/l | 3 | 1 | 0 | 0 |
| Use of vaso-active drugs | 3 | 6 | 3 | 2 |
| New barotrauma | 1 | 0 | 0 | 0 |

Comparison between the number of patients with hemodynamic instability and new barotrauma before and after the lung volume optimization maneuver. No significant differences were observed. Data are depicted as absolute number. P10 tenth percentile; p90 ninetieth percentile.
